# Pregnancy-Related Stress Among Pregnant Women Receiving Tocolytic and Non-Tocolytic Treatments Where Both Used Complementary Medicine

**DOI:** 10.3389/fphar.2022.870659

**Published:** 2022-06-02

**Authors:** Chen-Yuan Hsu, Ching-Li Chen, Li-Yun Tsai, Jung-Mei Tsai

**Affiliations:** ^1^ College of Nursing and Health Sciences, Dayeh University, Dacun, Taiwan; ^2^ Taichung Veterans General Hospital, Taichung, Taiwan; ^3^ Mackay Memorial Hospital, Taipei, Taiwan; ^4^ Department of Nursing, Mackay Junior College of Medicine, Nursing, and Management, Taipei, Taiwan; ^5^ Department of Nursing, Mackay Medical College, New Taipei, Taiwan

**Keywords:** tocolysis, tocolytic, pregnant women, pregnancy stress, complementary medicine

## Abstract

**Objective:** This study aimed to compare the pregnancy stress among pregnant women in receiving tocolytic and non-tocolytic treatments where both used complementary medicine.

**Methods:** A cross-sectional survey was conducted among 35 pregnant women receiving tocolytic treatment and 35 receiving non-tocolytic treatment, where both used complementary medicine in a medical center in central Taiwan. A basic information questionnaire that contained demographic variables and types of complementary medicine used and the Pregnancy Stress Rating Scale were used for the analysis.

**Results:** The types of complementary medicines were surveyed using the multiple-choice questionnaire. Natural products (77.5%) were most commonly used by pregnant women receiving tocolytic treatment, followed by alternative medicine (13.75%), manipulative and body-based practices (5%), and mind and body medicine (3.75%). In pregnant women who were receiving non-tocolytic treatment, natural products (59.1%) were most commonly used, followed by manipulative and body-based practices (16.4%), alternative medicine (15.4%), mind and body medicine (7.3%), and energy therapy (1.8%). According to the analysis of covariance test results, while both used complementary medicine in groups, pregnant women receiving tocolytic treatment were less stressed than those who were receiving non-tocolytic treatment (Pregnancy Stress Rating Scale score, *p* = 0.038), especially in dimension 2 (stress caused by infant care and changes in family relationships) (*p* = 0.015) and dimension 5 (stress caused by changes in physical appearance and function) (*p* = 0.008), which showed statistically significant differences (*p* < 0.05). Linear regression analysis results showed that the gestational age significantly associated with pregnancy stress (Pregnancy Stress Rating Scale score, *p* = 0.029; dimension 2, *p* = 0.016; and dimension 5, *p* = 0.001).

**Conclusion:** Among both who used complementary medicine, pregnancy stress was significantly lower in pregnant women who were receiving tocolytic treatment than in those who were receiving non-tocolytic treatment. This finding can be used as a reference for future pregnant women’s health studies.

## 1 Introduction

Physiological, psychological, social, or fetal factors may be detrimental to prenatal health, possibly leading to miscarriages, premature birth, or stillbirths (Hu, 2006; Dhillon et al., 2017). Thus, pregnant women may experience pregnancy stress; to reduce or eliminate such stress, they resort to using complementary medicine. Complementary medicine, such as religion, music, or acupuncture can reduce anxiety, unease, and uncertainty in tocolytic (a category of drugs used to delay the labor process) pregnant women and increase the therapeutic effectiveness of tocolysis (Tsai and Lee, 2012). Given these findings, complementary medicine and pregnancy stress are worthy to be investigated.

Complementary medicine has diverse types but is generally divided into five major categories: alternative medicine, natural products, mind and body medicine, energy therapy, and manipulative and body-based practices. Alternative medicines such as traditional Chinese medicine and naturopathy are slow-acting but can improve chronic diseases (Chiang, 2014; Pallivalappila et al., 2014; Mitchell, 2016). A 2008 survey involving obstetricians from the American Medical Association considered biofeedback, chiropractic care, acupuncture, and meditation as highly effective complementary medicine treatments (Babbar et al., 2017). Natural products emphasize that the human body itself has natural healing capabilities and that natural substances, such as natural herbs, high doses of vitamins, minerals, dietary fiber, probiotics, Lingzhi mushroom, shark cartilage, and cod liver oil, can be used to prevent diseases (Holst et al., 2009). In mind and body medicine, psychological stability can be improved using certain techniques, such as imagery, meditation, music therapy, yoga, prayer, biofeedback, journal therapy, art therapy, sitting still, humor, Tai Chi, and psychotherapy. Energy therapies, such as healing touch, therapeutic touch, Reiki, Qigong, and magnet therapy, refer to using energy from certain substances to stimulate the human body to achieve health. Last, manipulative and body-based practices are based on body manipulation and body movements, and these include chiropractic medicine, various massage techniques, body movements (e.g., osteopathy), rolfing, light therapy, hydrotherapy, and chromotherapy (Chen et al., 2010; Birdee et al., 2014; Guardino et al., 2014).

Currently, many pregnant women receiving tocolytic treatment use complementary medicine at suitable times to alleviate prenatal psychological stress and reduce stress, anxiety, labor pain, and depression, especially during labor. Hence, complementary medicine provides therapeutic effects during the entire pregnancy period (Deligiannidis and Freeman, 2014). Some types of complementary medicines are also specifically effective in mental disorders (Barcelona de Mendoza et al., 2016; Smith et al., 2019). However, pregnancy stress varies from individual to individual. The severity and type of stress experienced at the same pregnancy stage still varies because women differ in individual factors and life events. Stress occurs when pregnant women feel that they lose control or unable to cope (Yang and Chung, 2009). Rubin believes that maternal roles develop during pregnancy; one role is ensuring a healthy fetomaternal state until delivery (Rubin, 1976).

Frequently, pregnant women receiving tocolytic treatment possess factors that threaten maternal and fetal health, leading to difficulty in adapting to maternal roles (Rubin, 1976). This event causes pregnancy stress, which worsens if the pregnancy outcomes are uncertain; hence, pregnant women need more social support to adapt to pregnancy, particularly from their husbands and other family members. To alleviate such stress and uncertainty, using complementary medicine with pregnant women can be taught to use research-validated relaxation techniques, such as music, breathing techniques, and art therapy (Fang et al., 2011).

In Taiwan, most studies on complementary medicine treatment during pregnancy focused on the effectiveness of a certain type of complementary medicine, such as mind and body medicine (Mitchell, 2016). In addition, pregnancy stress in pregnant women receiving tocolytic treatment who use complementary medicine remains unreported. Therefore, this study aimed to compare pregnancy stress between pregnant women receiving tocolytic treatment and those who were receiving non-tocolytic treatment, where both used complementary medicine, to provide a reference for future studies by clinical staff.

## 2 Materials and Methods

### 2.1 Study Design

This study is a cross-sectional survey that used convenience sampling from January 2019 to April 2019. After collecting data using structured questionnaires, we compared pregnancy stress among pregnant women receiving tocolytic treatment and those who were receiving non-tocolytic treatment, where both used complementary medicine. In this study, pregnant women receiving tocolytic treatment were defined as those within 20 and 37 gestational weeks undergoing tocolysis because of the risk of placenta previa, premature labor, or premature rupture of membrane. Conversely, pregnant women who were receiving non-tocolytic treatment were defined as those within 20–40 gestational weeks who were not prone to premature birth or had high-risk pregnancy and did not undergo tocolysis.

### 2.2 Study Site and Subjects

We recruited pregnant women receiving tocolytic treatment and those who were receiving non-tocolytic treatment admitted to the delivery room, obstetric ward, and obstetric outpatient unit of Taichung Veterans General Hospital in central Taiwan. The inclusion criteria were women with confirmed pregnancy; with and without tocolysis; who could listen, speak, read, and write Chinese and communicate in Chinese or Taiwanese Hokkien; and who were willing to participate in this study. The exclusion criteria were a history of mental illness, impaired consciousness, or communication disorder.

Pregnant women who use the following as prescribed by the medical center as kind of tocolytics and doses (drug treatments are from the lowest to the highest dose, and the dose was adjusted according to the maternal fetus condition) were recruited in receiving the tocolytic treatment group. 1) Drug name: nifedipine/5 gm, it is advised to take one to two tablets orally, once every 4 or 6 hours or to use when necessary (PRN, pro re nata/as necessary). 2) Drug name: Yutopar (atosiban) 250 mg/amp, 5 amp, normal saline (N/S) (225 ml) is added and maintained at 3–21 ml per hour. 3) Drug name: MgSO_4_ (magnesium sulfate) maintained at 1–2 g per hour. 4) Drug name: atosiban two vials normal saline (N/S) (90 ml) were added and maintained at 2–12 ml per hour. Pregnant women who did not receive tocolytic treatment were recruited in the non-tocolytic treatment group.

### 2.3 Rights and Ethical Considerations of the Study Subjects

This study was reviewed and approved by the Institutional Review Board of the hospital (IRB NO: CE18194A). Data collection officially began after fulfilling the administrative procedures and obtaining written informed consent from all study participants.

### 2.4 Study Tools

This study employed structured questionnaires to collect patient data. The study tools included a basic information questionnaire and the Pregnancy Stress Rating Scale (Chen, 2015), which are briefly summarized as follows:

#### 2.4.1 Basic Information Questionnaire

This questionnaire was created by ourselves, and it includes demographic variables such as last menstrual period (LMP) to understand the gestational age, age, occupation, marital status, education level, religious beliefs, gravidity, and tocolysis experience (e.g., women with a history of tocolysis in previous pregnancy), as well as a survey on the types of complementary medicine used.

#### 2.4.2 Pregnancy Stress Rating Scale

We used the Pregnancy Stress Rating Scale (Chen, 2015) to evaluate pregnancy stress. It contains five dimensions as follows: dimension 1 (stress caused by ensuring mother and child health and safety, with nine questions); dimension 2 (stress caused by infant care and changes in family relationships, with nine questions); dimension 3 (stress caused by acknowledging maternal role, with eight questions), dimension 4 (stress caused by seeking social support, with four questions), and dimension 5 (stress caused by changes in physical appearance and function, with six questions). Each question was answered using a 5-point Likert scale (never, one point; occasionally, two points; sometimes, three points; often, four points; and always, five points). The higher the score, the greater will be the pregnancy stress. The validity and reliability tests of the scale (Chen, 2015) obtained *α* = 0.92 and can jointly achieve 52.17% of the total variance; thus, the scale is fairly valid and reliable.

### 2.5 Data Analysis

All statistical data were analyzed using the Statistical Package for the Social Sciences version 22.0. The demographic variables and usage distribution for complementary medicine were analyzed by descriptive statistics. Proportions were compared by the *χ* ^*2*^ test. For inferential statistics, the means between groups were compared by the analysis of covariance (ANCOVA) test. Furthermore, the predictive relationship between variables was evaluated by regression analysis. To estimate the sample size, we employed power analysis (Polit and Hungler, 1999), with >0.8 as the statistical power. When *α* (significance level) was set at 0.05, the power was 0.8, and the effect size was 0.4; thus, 35 subjects were required for each group. In consideration of the timeliness of subject enrollment and sample loss, 70 subjects were enrolled in all groups. The questionnaire was personally sent to the participants, and after the completion of all questions, no data were missed. The reliability of this research was assessed using Cronbach’s *α* to assess the internal consistency. Cronbach’s *α* was 0.92. For all statistical tests, *p* < 0.05 indicated significance.

## 3 Results

### 3.1 Distribution of the Basic Information of Pregnant Women Receiving Tocolytic and Non-tocolytic Treatments

Tocolysis usually occurs in the second trimester. In this study, the mean gestational age of pregnant women receiving tocolytic treatment and those who were receiving non-tocolytic treatment was 27.94 and 38.54 weeks, respectively. Most of the pregnant women were aged 20–34 years, homemaker, married, had a senior high school education or above, absent of religious beliefs, primigravida, and never had a history of tocolysis in previous pregnancy in both the groups. According to the *χ* ^*2*^ test results, the demographic variables were not significantly different between the two groups (Table 1).

**TABLE 1:** Analysis of demographic variables (*n* = 70).

Variable	Pregnant women receiving tocolytic treatment(*n* = 35) *n* (%)	Pregnant women receiving non-tocolytic treatment (*n* = 35) *n* (%)	*p*
Age			1.000
20–34 years	23 (65.7)	23 (65.7)	
35–45 years	12 (34.3)	12 (34.3)	
Occupation			0.271
Bureaucrat	2 (5.7)	6 (17.1)	
Farmer	0 (0)	1 (2.9)	
Artisan	5 (14.3)	4 (11.4)	
Merchant	12 (34.3)	6 (17.1)	
Homemaker	15 (42.9)	18 (51.4)	
Service industry	1 (2.9)	0 (0)	
Marital status			0.314
Married	34 (97.1)	35 (100)	
Unmarried	1 (2.9)	0 (0)	
Education level			1.000
Below senior high school	6 (17.1)	6 (17.1)	
Senior high school and above	29 (82.9)	29 (82.9)	
Religious beliefs			0.811
Present	17 (48.6)	16 (45.7)	
Absent	18 (51.4)	19 (54.3)	
Gravidity			0.803
Primigravida	22 (62.9)	23 (65.7)	
Multigravida	13 (37.1)	12 (34.3)	
Tocolysis experience (women with a history of tocolysis in previous pregnancy)			0.615
Yes	13 (37.1)	11 (31.4)	
No	22 (62.9)	24 (68.6)	

### 3.2 Analysis of the Types of Complementary Medicine Used by Pregnant Women Receiving Tocolytic and Non-tocolytic Treatments

**FIGURE 1:**
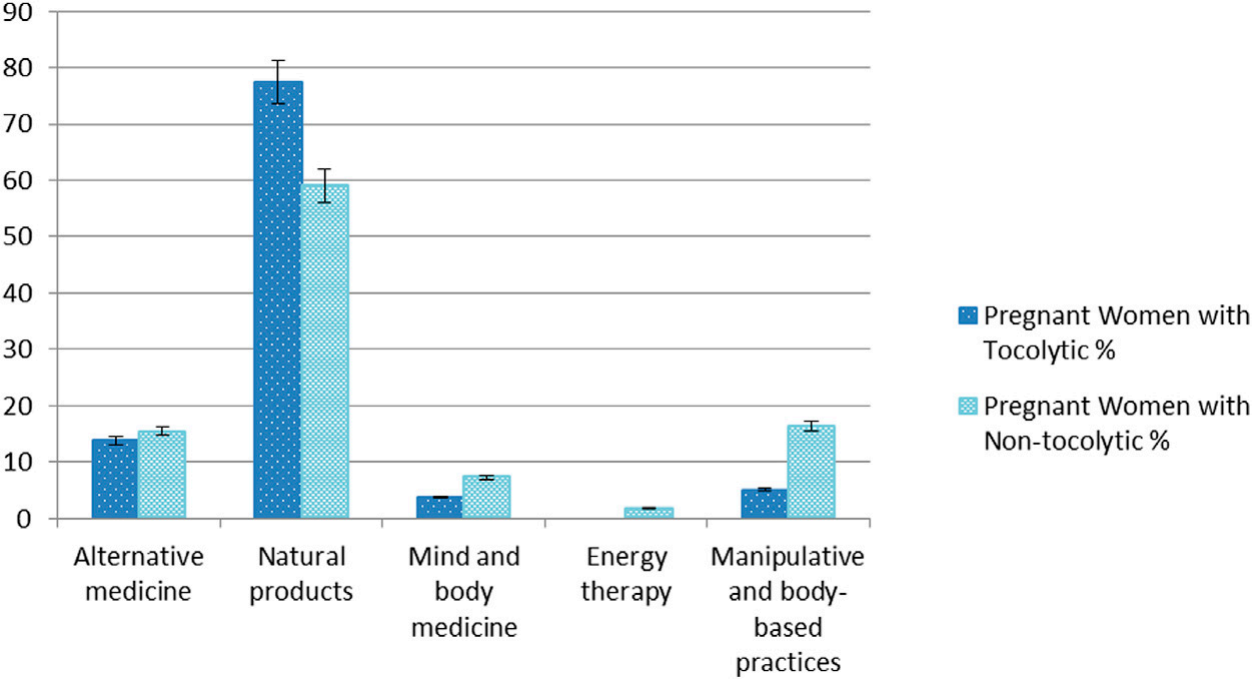
Types of complementary medicine used by pregnant women receiving tocolytic and non-tocolytic treatment.

The types of complementary medicine used during pregnancy were surveyed using the multiple-choice questionnaire, and the results were calculated according to the number of subjects. Natural products (*n* = 62, 77.5%) were most commonly used by pregnant women receiving tocolytic treatment, followed by alternative medicine (*n* = 11, 13.75%), manipulative and body-based practices (*n* = 4, 5%), and mind and body medicine (*n* = 3, 3.75%). In pregnant women who were receiving non-tocolytic treatment, natural products (*n* = 65, 59.1%) were most commonly used, followed by manipulative and body-based practices (*n* = 18, 16.4%), alternative medicine (*n* = 17, 15.4%), mind and body medicine (*n* = 8, 7.3%), and energy therapy (*n* = 2, 1.8%). The natural products used by these pregnant women mainly consisted of supplements, such as various vitamins, probiotics, and cod liver oil; meanwhile, alternative medicine, manipulative and body-based practices, and mind and body medicine were mainly traditional Chinese medicine, massage, and yoga, respectively. According to the χ2 test results, the types of complementary medicine used during pregnancy were not significantly different between the two groups (Table 2; Figure 1).

**TABLE 2:** Analysis of the types of complementary medicine used by pregnant women receiving tocolytic and non-tocolytic treatment (*n* = 70).

Variable (multiple-choice)	(*n*)	Pregnant women receiving tocolytic treatment *n* (%)	(*n*)	Pregnant women receiving non-tocolytic treatment *n* (%)	*p*
Alternative medicine		11 (13.75)		17 (15.4)	0.069
Traditional Chinese medicine	6		12		
Acupuncture	2		1		
Moxibustion	1		0		
Chinese herbal medicine	1		3		
Tuina	1		0		
Tai chi	0		0		
Qigong	0		1		
Natural products		62 (77.5)		65 (59.1)	0.088
Natural herbs	2		0		
Vitamins	24		28		
Minerals	4		5		
Dietary fiber	6		9		
Probiotics	18		13		
Cod liver oil	8		10		
Mind and body medicine		3 (3.75)		8 (7.3)	0.101
Meditation	0		3		
Yoga	2		4		
Hypnosis	1		0		
Progressive relaxation and guided imagery	0		1		
Energy therapy		0 (0)		2 (1.8)	0.151
Artificial magnet	0		1		
Natural magnet	0		1		
Manipulative and body-based practices		4 (5)		18 (16.4)	0.162
Osteopathy	0		1		
Massage	4		17		

### 3.3 Comparison of Pregnancy Stress Among Pregnant Women Receiving Tocolytic and Non-tocolytic Treatments Where Both Used Complementary Medicine

The groups were compared by the ANCOVA test to determine the pregnancy stress between pregnant women receiving tocolytic and non-tocolytic treatments where both used complementary medicine. The influence of covariates (i.e. gestational age) was also examined on the pregnancy stress has been adjusted for gestational age as a confounder. The Pregnancy Stress Rating Scale score was significantly (*p* = 0.038) higher in pregnant women who were receiving non-tocolytic treatment (Mean ± SD: 92.17 ± 21.61) than in their tocolytic (Mean ± SD: 81.74 ± 17.20) counterparts where both used complementary medicine. Dimension 2 (stress caused by infant care and changes in family relationships) between the pregnant women with the tocolytic group (Mean ± SD: 16.91 ± 4.49) and the non-tocolytic group (Mean ± SD: 20.40 ± 6.00) showed statistically significant differences (*p* = 0.015). Dimension 5 (stress caused by changes in physical appearance and function) between the pregnant women with the tocolytic (Mean ± SD: 14.40 ± 4.76) and non-tocolytic groups (Mean ± SD: 18.34 ± 6.14) showed statistically significant differences (*p* = 0.008). Whereas other dimensions, revealed no significant differences (Table 3 **)**. Both the groups used complementary medicine, and pregnancy stress was significantly lower in pregnant women who were receiving tocolytic treatment than those who were receiving non-tocolytic treatment.

**TABLE 3:** Comparison of pregnancy stress among pregnant women receiving tocolytic and non-tocolytic treatment where both used complementary medicine (*n* = 70).

Pregnancy Stress Rating Scale	Pregnant women receiving tocolytic treatment (*n* = 35) (mean ± SD)	Pregnant women receiving non-tocolytic treatment (*n* = 35) (mean ± SD)	*F*	*p*
Pregnancy Stress Rating Scale score	81.74 ± 17.20	92.17 ± 21.61	3.43	0.038^a^
Dimension 1: stress caused by ensuring mother and child health and safety	23.71 ± 6.52	25.00 ± 8.38	1.81	0.171
Dimension 2: stress caused by infant care and changes in family relationships	16.91 ± 4.49	20.40 ± 6.00	4.48	0.015^a^
Dimension 3: stress caused by acknowledging the maternal role	20.14 ± 4.52	21.31 ± 4.33	1.32	0.294
Dimension 4: stress caused by seeking social support	6.57 ± 2.48	7.11 ± 2.96	1.51	0.227
Dimension 5: stress caused by changes in physical appearance and function	14.40 ± 4.76	18.34 ± 6.14	5.15	0.008^a^

a *p* < .05.

SD, standard deviation.

### 3.4 Gestational Age and Pregnancy Stress

Linear regression analysis results revealed that the gestational age significantly associated pregnancy stress according to the Pregnancy Stress Rating Scale score (*p* = 0.029), especially in dimension 2 [stress caused by infant care and changes in family relationships (*p* = 0.016)] and dimension 5 [stress caused by changes in physical appearance and function (*p* = 0.001)]. Regarding its predictive ability, gestational age obtained 6.8% for the overall Pregnancy Stress Rating Scale score, 8.2% for dimension 2, and 15.4% (R2) for dimension 5. Thus, dimensions 2 and 5 explained the gestational age associated with stress in those categories (Table 4).

**TABLE 4:** Gestational age and pregnancy stress (*n* = 70).

Pregnancy Stress Rating Scale	Gestational age			
	β	*R* ^2^	*p*	95% Confidence interval
Pregnancy Stress Rating Scale score	0.261	0.068	0.029^a^	0.086–1.52
Dimension 1: stress caused by ensuring mother and child health and safety	0.127	0.016	0.296	−0.130–0.422
Dimension 2: stress caused by infant care and changes in family relationships	0.286	0.082	0.016^a^	0.046–0.442
Dimension 3: stress caused by acknowledging the maternal role	0.101	0.010	0.406	−0.096–0.233
Dimension 4: stress caused by seeking social support	−0.003	0.000	0.978	−0.103–0.100
Dimension 5: stress caused by changes in physical appearance and function	0.392	0.154	0.001^a^	0.151–0.549

a *p* < .05.

## 4 Discussion/Conclusion

The distribution of basic information between the pregnant women receiving tocolytic treatment and those who were receiving non-tocolytic treatment in this study was similar to that in a previous research involving pregnant women in Taiwan (Holst et al., 2009); most of the pregnant women aged 20–34 years, married, had a senior high school education or above, and primigravida. The present study then discussed and compared in the usage of complementary medicine and pregnancy stress between pregnant women receiving tocolytic treatment and those who were receiving non-tocolytic treatment. The findings can serve as future care guidelines and research directions. The insights into whether gestational age, preterm birth risk, and uncertainty can increase pregnancy stress should also be understood in detail. Pregnant women require sufficient time to rest, with a more accurate understanding of current complementary medicine usage during pregnancy through interviews, and environmental interference factors can be reduced.

This study analyzed the types of complementary medicine commonly used by pregnant women (Chiang, 2014; Pallivalappila et al., 2014; Mitchell, 2016) and found five major categories: alternative medicine, natural products, mind and body medicine, energy therapy, and manipulative and body-based practices. The complementary medicine types were reported between the groups of pregnant women receiving tocolytic treatment and those who were receiving non-tocolytic treatment. Both groups most frequently used natural products, the second most commonly used approach was alternative medicine in pregnant women receiving tocolytic treatment, and manipulative and body-based practices in those who were receiving non-tocolytic treatment. The study results clearly demonstrated those pregnancy characteristics among pregnant women, the choice and application of complementary medicine. As Sheraton et al. (2018) reported that women are increasingly using complementary and alternative therapies during pregnancy. This view is worthy of further investigation.

Regarding the use of alternative medicine during pregnancy, traditional Chinese medicine was most commonly used, followed by acupuncture, Chinese herbal medicine, and Tuina, similar to a study in New Zealand (Zheng et al., 2018). Both groups mostly used vitamins, probiotics, and cod liver oil as natural products, similar to previous studies (Johnson et al., 2016; Gilmartin et al., 2018; Zheng et al., 2018). Moreover, yoga and hypnosis were mainly used as mind and body medicine, consistent with previous studies (Chen et al., 2013; Gavin et al., 2020). In this study, pregnant women receiving tocolytic treatment did not use energy therapy, and no literature study has reported the use of energy therapy during pregnancy. In pregnant women who were receiving non-tocolytic treatment, massage was mostly used as a manipulative and body-based practice, similar to previous studies (Hung and Chiang, 2017; Fogarty et al., 2019).

The Pregnancy Stress Rating Scale score, dimension 2 (stress caused by infant care and changes in family relationships), and dimension 5 (stress caused by changes in physical appearance and function) were significantly different between the two patient groups. Although both groups used complementary medicine, the pregnant women receiving tocolytic treatment showed less pregnancy stress than those who were receiving non-tocolytic treatment. Dimension 2, which focuses on stress caused by infant care and changes in family relationships, was significantly lower in pregnant women receiving tocolytic treatment than those who were receiving non-tocolytic treatment. Family relationship changes require important family support to achieve the entire process of adaptation. Therefore, the acceptance of others during pregnancy is very important to the psychology of pregnancy, such as pregnant women receiving tocolytic treatment who just focus on a fetus, hope to ensure the safety of themselves and their fetus (Chen, 2015; Chang et al., 2016).

Dimension 5, which refers to stress caused by changes in physical appearance and function, showed the same finding. The pregnancy stress can be caused by changes in body appearance and function becomes more obvious as the pressure increases during pregnancy (Chen, 2015). Therefore, we hypothesized that as the gestational age advances in pregnant women, their physical appearance changes more significantly. This view is consistent with a previous study (Chang et al., 2016), which reported that stress caused by changes in physical appearance and function appears in early pregnancy and gradually peaks at late pregnancy.

However, the Pregnancy Stress Rating Scale scores for dimension 1 (stress caused by ensuring mother and child health and safety), dimension 3 (stress caused by acknowledging maternal role), and dimension 4 (stress caused by seeking social support) were not significantly different between the two groups. These dimensions are critical for both pregnant women receiving tocolytic and those who were receiving non-tocolytic treatment, consistent with Rubin’s view that maternal tasks develop during pregnancy. Examples for such tasks include ensuring that the mother and fetus can successfully undergo pregnancy and delivery and adapt to maternal roles (Rubin, 1976). This result is consistent with a previous study (Fang et al., 2011). Thus, pregnant women require more social support to adapt to pregnancy. Generally, pregnant women receiving tocolytic treatment have factors that are detrimental to maternal and fetal health, resulting in difficulty in overcoming pregnancy-related stress.

Mental health should be considered during pregnancy (Gong et al., 2020; Martínez-Borba et al., 2020; Rodríguez-Muñoz et al., 2020). The present study revealed pregnancy stress. However, convenience sampling limits the generalizability of the findings. We collected data from only one medical center in central Taiwan. Therefore, generalizing the results to other pregnant women receiving tocolytic and those who were receiving non-tocolytic treatment should be undertaken with caution.

This study provides the following recommendations for future research directions, education, and clinical practice. First, regarding future research, healthcare professionals should thoroughly examine community homes to explore the correlation between pregnant women receiving tocolytic treatment who use complementary medicine and their pregnancy stress. They must ensure that they understand the types of complementary medicine and their effects in such people. Regarding educational recommendations, healthcare professionals should develop knowledge and skills related to complementary medicine to improve their care ability for pregnant women receiving tocolytic treatment. In clinical settings, they can explore and understand how to provide comprehensive care for pregnant women receiving tocolytic treatment and to know the pregnancy stress of pregnant women.

In conclusion, although both pregnant women receiving tocolytic treatment and those who were receiving non-tocolytic treatment used complementary medicine, pregnancy stress was significantly lower in the former than in the latter. This finding can be used as a reference for future studies on pregnant women’s health. All findings in this study support the use of complementary medicine at suitable times if pregnant women agree that complementary medicine is beneficial during pregnancy.

## Data Availability

The original contributions presented in the study are included in the article/Supplementary Material, further inquiries can be directed to the authors.
