# Corrigendum: Pregnancy-related stress among pregnant women receiving tocolytic and non-tocolytic treatments where both used complementary medicine

**DOI:** 10.3389/fphar.2022.979142

**Published:** 2022-08-05

**Authors:** Chen-Yuan Hsu, Ching-Li Chen, Li-Yun Tsai, Jung-Mei Tsai

**Affiliations:** ^1^ College of Nursing and Health Sciences, Dayeh University, Dacun, Taiwan; ^2^ Taichung Veterans General Hospital, Taichung, Taiwan; ^3^ Mackay Memorial Hospital, Taipei, Taiwan; ^4^ Department of Nursing, Mackay Junior College of Medicine, Nursing and Management, Taipei, Taiwan; ^5^ Department of Nursing, Mackay Medical College, New Taipei, Taiwan

**Keywords:** tocolysis, tocolytic, pregnant women, pregnancy stress, complementary medicine

In the published article, there was an error in the Ethical Statement (IRB NO).

A correction has been made to **2.3 Rights and Ethical Considerations of the Study Subjects Name of Section**, first sentence. The corrected sentence appears below:

“This study was reviewed and approved by the Institutional Review Board of the hospital (IRB NO: CE18194A).”

A correction has been made to **Ethics Statement**, first sentence. The corrected sentence appears below:

“The studies involving human participants were reviewed and approved by the regional ethics committee of the Institutional Review Board of Taichung Veterans General Hospital, Taichung, Taiwan (IRB NO: CE18194A).”

The authors apologize for this error and state that this does not change the scientific conclusions of the article in any way. The original article has been updated.

